# Faster decline of pitch memory over time in congenital amusia

**DOI:** 10.2478/v10053-008-0073-5

**Published:** 2010-04-26

**Authors:** Victoria J. Williamson, Claire McDonald, Diana Deutsch, Timothy D. Griffiths, Lauren Stewart

**Affiliations:** ^1^Department of Psychology, Goldsmiths, University of London, UK; ^2^Psychology Department, University of California, La Jolla, CA, USA; ^3^Institute of Neuroscience, Newcastle University, Newcastle upon Tyne, UK

**Keywords:** congenital amusia, short-term memory, delay, tonal interference

## Abstract

Congenital amusia (*amusia*, hereafter) is a developmental disorder that impacts negatively on the perception of music. Psychophysical testing suggests that individuals with amusia have above average thresholds for detection of pitch change and pitch direction discrimination; however, a low-level auditory perceptual problem cannot completely explain the disorder, since discrimination of melodies is also impaired when the constituent intervals are suprathreshold for perception. The aim of the present study was to test pitch memory as a function of (a) time and (b) tonal interference, in order to determine whether pitch traces are inherently weaker in amusic individuals. Memory for the pitch of single tones was compared using two versions of a paradigm developed by Deutsch (1970a). In both tasks, participants compared the pitch of a standard (S) versus a comparison (C) tone. In the time task, the S and C tones were presented, separated in time by 0, 1, 5, 10, and 15 s (blocked presentation). In the interference task, the S and C tones were presented with a fixed time interval (5 s) but with a variable number of irrelevant tones in between 0, 2, 4, 6, and 8 tones (blocked presentation). In the time task, control performance remained high for all time intervals, but amusics showed a performance decrement over time. In the interference task, controls and amusics showed a similar performance decrement with increasing number of irrelevant tones. Overall, the results suggest that the pitch representations of amusic individuals are less stable and more prone to decay than those of matched non-amusic individuals.

## Introduction

The requisite skills that allow adults to engage with and respond to musical sounds are acquired effortlessly in early life in all known cultures of the world, without the need for formal instruction (Trehub & Hannon, 2006). However, up to 1 in 25 individuals may be born with a developmental disorder termed *congenital amusia* (henceforth *amusia*) which impacts negatively on music processing, despite a normal amount of musical exposure, normal hearing ability, and no concomitant intellectual or neurological impairments (Ayotte, Peretz, & Hyde, 2002; Kalmus & Fry, 1980; Peretz et al., 2002). Singing ability is often impaired in amusia, however, amusic individuals should be distinguished from the larger self-report population who describe themselves as *tone deaf* (Cuddy et al., 2005; Sloboda et al., 2005; Wise & Sloboda, 2008). The primary deficit in amusia is thought to be associated with difficulties in the perception of music, which then have secondary consequences for production.

It is currently possible to examine the extent of music perception problems in amusia due to the development the Montreal Battery for the Evaluation of Amusia (MBEA), a standardised diagnostic tool which requires discrimination of melodies where a single tone may differ based on one of the major musical attributes of the tune, such as key, contour (patterns of ups and downs), or pitch height (Peretz, Champod, & Hyde, 2003). Psychophysical tests of amusic individuals diagnosed using the MBEA found that they had higher thresholds for the detection of a pitch change and discrimination of pitch direction (Foxton, Dean, Gee, Peretz, & Griffiths, 2004; Hyde & Peretz, 2004). Such a low-level disorder of auditory perception could conceivably explain poor scores on the MBEA, however, not all individuals with amusia show elevated thresholds and recent research has indicated that there may also be an ancillary deficit in short-term memory. Both Foxton et al. (2004) and Tillmann, Schulze, and Foxton (2009) reported that individuals with amusia had difficulty discriminating pitch sequences when the constituent intervals exceeded psychophysical thresholds for the detection of a pitch change. Tillmann et al. compared recognition of five item sequences which comprised tones, words, or timbres. Amusics performed similarly to controls on the word sequences but were significantly worse for tone (*p* < .001) and timbre sequences (*p* = .04), indicating that amusics’ poor performance on short-term sequence recognition may be specific to music-like stimuli and most apparent with manipulations of pitch.

While Tillmann et al. (2009) considered memory for sequences of tones in amusia much is still unknown concerning the fidelity with which amusics store pitch information for single tones. A previous study (Gosselin, Jolicoeur, & Peretz, 2009) compared amusic and control performance on a task requiring comparison of two tones, separated by a silent pause (1650 ms) or a pause of the same length filled with irrelevant tones. While controls showed good performance in both cases with pitch distances of 1, 2, and 3 tones, amusics were barely above chance for either condition at a pitch distance of 1 tone. For pitch distances of 2 and 3 tones, performance was good for the silent condition but significantly worse when irrelevant tones were present.

The afore-mentioned study indicates a deficit in the storage of pitch for single tones in amusia and provides a rationale for further investigation into this issue. For instance, the previous study does not reveal the extent to which memory representations decay with time, or with an increasing amount of interference. Previous literature from non-amusic participants has demonstrated contrasting effects of time and interference on memory for auditory material. In the absence of interference, representations show relatively little decay, even up to 10 s (Clement, Demany, & Semal, 1999; Harris, 1952; Lewandowsky, Oberauer, & Brown, 2009) but the presence of even a single tone during the retention interval impairs performance (Elliot, 1970; Massaro, 1970) with additional tones exacerbating this performance decrement (Deutsch, 1970b).

This literature on time and interference effects in non-amusic individuals, coupled with the interesting findings of Gosselin et al. (2009) , motivated the current study in which we use a standard tone comparison paradigm (Deutsch, 1970a, 1973, 1978) to investigate amusics’ ability to (a) maintain a representation of pitch with increasing time (*time task*) and (b) maintain a representation of pitch at a fixed time with increasing interference (*interference task*).

## Method

### Design

Both tasks in the present study (time or interference task) had a 2 x 5 split-plot design. The between-subject variable was group (amusic and control) and each within-subject variable had five levels; either inter-stimulus intervals of 0, 1, 5, 10, and 15 s (time task) or number of interpolated tones, specifically 0, 2, 4, 6, and 8 (interference task).

### Participants

Thirty-four participants (17 amusic) took part in the time task and 32 (16 amusic) in the interference task, in return for a small honarium. All participants first completed an online assessment that was based on the scale subtest of the Montreal Battery for the Evaluation of Amusia (MEBA; Peretz et al., 2003; see www.delosis.com/listening/home.html). Participants took the online test twice and if they consistently achieved a score of 22/30 or less, they were invited to the laboratory to take the scale, contour, interval, and rhythm subtests of the MBEA under controlled conditions. Previous research has shown that amusia is characterized by poor performance on the pitch-based subtests of the MBEA (scale, contour, interval) while scores on the rhythm subtest are likely to be in the normal range for 50% of amusics (Peretz et al., 2003). For this reason, we calculated a composite score for the three pitch-based subtests, using 65 as a cut-off score (the sum of the cut-off scores for the three subtests in Peretz et al. (2003) ; those with composite scores below 65 were confirmed as amusic).

For the time task, 17 amusics were matched to 17 controls on gender, age, score on the National Adult Reading Test (a measure that correlates well with general intelligence) and digit span as measured by the Weschler Adult Intelligence Scale (WAIS-R). A summary of averages is presented in Table 1. All participants were also asked to rate their musical experience on a scale of 1-6 (see Appendix A). Nine amusics and 7 controls rated themselves as 5 on the scale indicating they had received no formal musical training. Five amusics and 7 controls rated themselves as 4, indicating a small amount of training at some point in their lives (amusics: *M* = 3.7 years; controls: *M* = 3.5 years).

**Table 1.** Participant Details (Time Task).

	Group	Age	NART	Digit span	MBEA scale	MBEA contour	MBEA interval	Pitch composite
μ	Amusic	48,41	42,24	20,94	18,18	18,94	17,71	54,82
σ		11,02	4,16	3,58	2,48	3,09	1,93	5,79
μ	Control	46,65	43,84	21,06	27,47	28,06	27,82	78,35
σ		11,98	3,10	3,19	2,03	1,98	2,10	20,75
*t*-test		-.44	1,28	.10	11,94	10,23	14,64	4,50
*p* value		.66	.21	.92	< **.001**	< **.001**	< **.001**	< **.001**

**Table 2.** Participant Details (Interference Task).

	Group	Age	NART	Digit span	MBEA scale	MBEA contour	MBEA interval	Pitch composite
μ	Amusic	51,00	41,25	19,38	18,19	18,50	17,19	53,88
σ		10,53	9,92	4,01	2,37	2,31	1,64	4,90
μ	Control	50,06	43,81	20,31	26,88	27,13	26,81	80,81
σ		10,04	5,53	4,90	1,54	1,41	2,01	4,25
*t*-test		-.26	.90	.59	12,28	12,75	14,85	16,62
*p* value		.80	.37	.56	< **.001**	< **.001**	< **.001**	< **.001**

The interference task was carried out in a different testing session, due to time constraints, and involved 16 amusics and 16 matched controls. The second group did not differ from the first on any of the variables, as can be seen in Tables 1 and 2. A total of 9 amusics and 2 controls took part in both tasks.

### Materials and procedure

#### Materials

Participants heard a standard tone (S), followed by a comparison tone (C), and were required to report whether the S and C tones were the same or different, saying “different” only if they were sure that a change had occurred. Same and different trials occurred equiprobably. When different the C tone was a whole tone higher or lower than the S tone. Psychophysical tests have shown that amusics have thresholds for the detection of a pitch change that are well below 1 semitone (Foxton et al., 2004).

In the time task, S and C tones were separated in time: 0 s (baseline), 1, 5, 10, and 15 s blocked across conditions. In the interference task, S and C tones were separated by a fixed interval of 5 s, but with intervening tones in between: 0 (baseline), 2, 4, 6, and 8 tones blocked across condition. Tones, generated in Matlab (http://www.mathworks.com/products/matlab/) were all pure tones, 200 ms in duration, with pitches taken from an equal tempered, chromatic scale, centered on the octave C4 and C5 (time task) or A4 and A5 (interference task). All pitches were used equally often within each condition, either as an S tone, a C tone, or both. In the interference task, distractor tones were chosen randomly, with the constraint that no sequence contained repeated tones. There was a 300 ms interval between the S tones and the first distractor tone, as well as between any consecutive distractor tones. Given that S and C tones were always 5 s apart this resulted in a pause between the final distractor tone and the C tone, as in Deutsch (1970a, 1973, 1978). Thus the distractor tones were not equally spaced throughout the 5 s interval and participants were made aware that there would be a pause following the distractor tones before presentation of the C tone. A schematic diagram was shown to participants to clarify the time line of events.

#### Procedure

In both the time and interference tasks, each condition comprised 24 trials, and commenced with 2 practice trials. Participants were told that they would hear 2 tones separated in time by a silent pause (time task), or by a fixed time interval containing a sequence of interpolated tones (interference task). Participants were asked to judge whether the first (S) and last (C) tones were exactly the same or different in pitch, ignoring any other interpolated tones. At the start of each trial participants heard a female voice saying “ready” to indicate that a trial was imminent. After the offset of the C tone participants immediately responded by saying “same” or “different” and their response was entered into the computer. There was a 10 s inter-trial interval. Each task took an average of 50 min to complete.

## Results

Performance on both tasks was scored using signal detection theory (Green & Swets, 1966). There were four possible responses. A “hit” (H) occurred when the participant correctly identified the C tone as different, whereas a miss would result from reporting “same” when the tones were different. When the C tone was the same as the S tone, participants scored a “correct rejection” if they reported “same” and a “false alarm” (FA) if they reported “different”. Scores were calculated using both a guess corrected (proportion of H/FA) and a *d*-prime formula (the difference between the *z*-transforms of the H and FA rates). No differences in the trends were found so only the guess corrected scores are reported.

**Figure 1.**
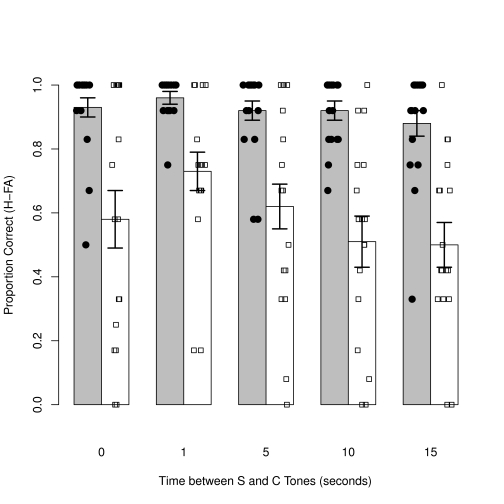
Average guess corrected scores in five conditions with increasing time delay between S and C tones. Error bars represent *SEM*.

### Time task

A 2 (Group) x 5 (Delay) ANOVA found a significant effect of group, indicating that controls performed better than the amusics overall, *F*(1, 32) = 21.1, *p* < .001, η_p_^2^ = .40 (.92 vs. .59). There was a significant detrimental effect of increasing delay, *F*(4, 128) = 6.5, *p* < .001, η_p_^2^ = .17, but no interaction with group, *F*(4, 128) = 2.2, *p* = .07.

Simple contrasts were carried out to compare the effects of increasing delay compared to the no-gap condition. The first contrast indicated the difference between the no-gap and one-second gap conditions was significant but in the direction opposite to that predicted; increasing delay lead to improved performance, *F*(1, 32) = 5.7, *p* = .02, η_p_^2^ = .15. This result had the potential to distort the general ANOVA, so a second analysis was carried out without the no-gap condition. This 2 x 4 ANOVA revealed the same patterns of main effect as the previous analysis, but the interaction between delay and group was significant, *F*(3, 96) = 4.0, *p* = .01, η_p_^2^ = .11. The nature of the interaction was investigated with corrected paired *t*-tests between the new baseline one-second gap condition and the other three delay conditions. The data shown in Table 3 suggest that an increase in the time between the S and C tones impaired the performance of amusics to a greater extent compared to controls.

**Table 3.** *T*-Test Analysis of Mean Difference Scores.

Group		1 vs. 5 s gap	1 vs. 10 s gap	1 vs. 15 s gap	5 vs. 10 s gap	10 vs. 15 s gap
Control	μ diff.	.04	.04	.08	.00	.03
	Σ	.89	.67	1,6	.09	.12
	*t*-value	2,05	2,73	2,02	< .001	1,33
	*p* value	.06	.02	.06	1,00	.20
Amusic	μ diff.	.11	**.22**	**.23**	.11	.01
	σ	.19	**.22**	**.21**	.20	.20
	*t*-value	2,28	*4,03*	*4,51*	2,26	.20
	*p* value	.04	.001*	<.001*	0.4	.84

*Note*. Mean difference scores between the baseline condition of 1 s, between S and C tone, and the three increasing delay conditions (5, 10, and 15 s), for amusic and control groups. Also included are comparisons between 5 and 10 s, and 10 and 15 s.). *Indicates significant at the .005 level.

### Interference task

There was a significant effect of group, indicating that controls performed better than the amusics overall, *F*(1, 30) = 59.1, *p* < .001, η_p_^2^ = .66 (.65 vs. .27). There was a significant detrimental effect of increasing number of interpolated tones, *F*(4, 120) = 51.7, *p* <.001, η_p_^2^ = .63, but no interaction between this factor and that of group, *F*(4, 120) = 0.4, *p* = .83. Although the two groups began the task at a different level of performance the analogous decline in performance for both groups indicates a similar proportional effect of interference as a result of increasing interpolated tones.

Due to the presence of a floor effect with 4 or more interpolated tones in the amusic group, we conducted an additional ANOVA comparing only the first two conditions where both groups were performing well above chance (0 interference and 2-tone interference). The pattern of results was the same; a significant main effect of adding interpolated tones compared to baseline, *F*(1, 30) = 19.9, *p* < .001, η_p_^2^ = .40, and no interaction with group, *F*(1, 30) = 0.3, *p* = .60, indicating that both controls and amusics showed a similarly detrimental effect of adding interpolated tones in the retention interval. We leave open the possibility that a group difference may have emerged in conditions where additional interpolated tones were added, had baseline performance levels been more similar across the two groups. However, in practice, a situation where baseline performance is matched across groups cannot be achieved without the use of different pitch intervals (between S and C tones) or a different interpolated time interval, between the two groups.

**Figure 2.**
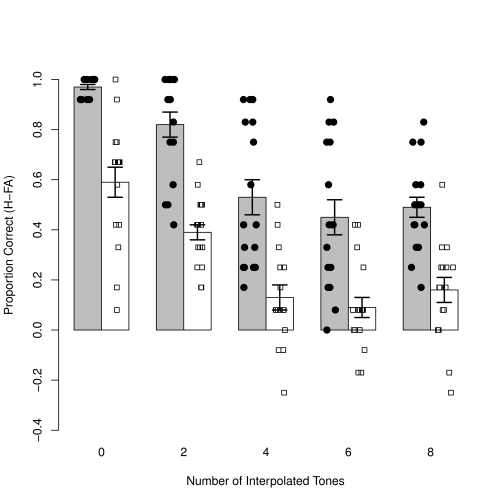
Average guess corrected scores in five conditions with increasing interference between S and C tones. Error bars represent *SEM*.

**Figure A1.**
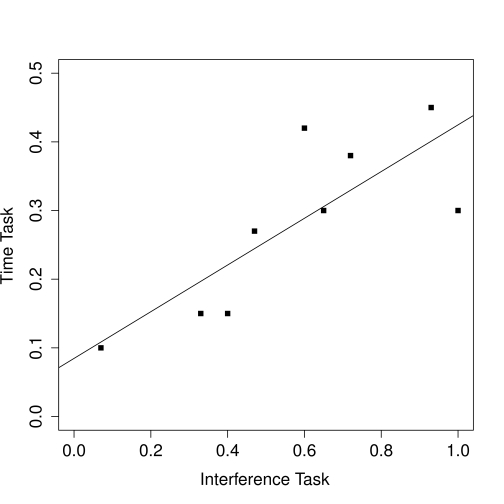
Scatterplot showing the significant positive correlation between composite performance on the time and interference tasks for 9 amusic participants (*r* = .80).

**Figure A2.**
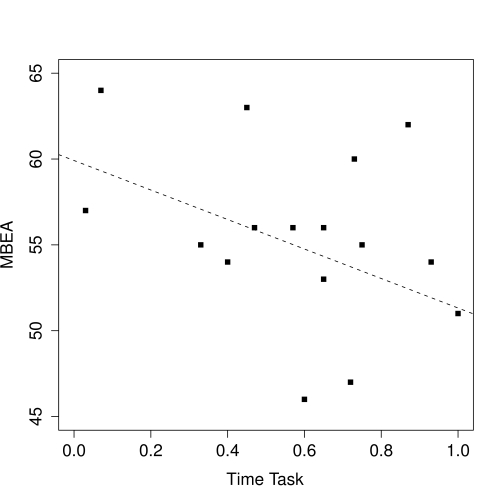
Scatterplot showing the null correlation between composite performances on the MBEA pitch tasks and the time task for all amusic participants (*r* = -.41).

**Figure A3.**
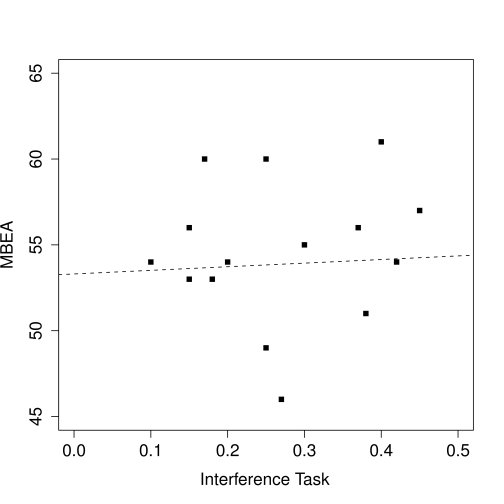
Scatterplot showing the null correlation between composite performances on the MBEA pitch tasks and the interference task for all amusic participants (*r* = .05).

Post hoc correlations were carried out on the time and interference task performance scores using data from the 9 amusics who had completed both parts of the experiment. Composite scores for each task were created by averaging performance across the five conditions and a significant positive relationship was found between the two sets of scores, *r* = .80, *p* = .01, suggesting that amusics who performed better on the time task were also likely to perform better on the interference task (see Figure A1). Correlations were also run between these 9 individuals’ MBEA pitch composite scores and their memory performance on both tasks, but this analysis found no significant relationships, implying that performance on the memory tasks utilized in the present experiment was not related to the severity of an individual’s amusia, as measured by the MBEA pitch composite score. This pattern of non-significant correlations remained when data from all other amusic participants was included in the analysis (see Figures A2 and A3).

## Discussion

The main aim of the present study was to extend our understanding of the cognitive deficits associated with congenital amusia. Past research has emphasised low-level auditory perceptual deficits (Foxton et al., 2004; Hyde & Peretz, 2004) but findings also suggest that deficits in pitch memory may play a role (Foxton et al., 2004; Tillmann et al., (2009). We investigated the nature of this pitch memory deficit, looking particularly at amusics’ ability to maintain the pitch of a sound in memory as a function of increasing time and auditory tonal interference.

In the time task the control group showed no impact of increasing delay upon their memory for a pitch sound. This finding is consistent with evidence that storage of sounds within auditory short-term memory deteriorates slowly in the absence of interference (Clement et al., 1999; Harris, 1952; Lewandowsky et al., 2009). Conversely, individuals with amusia showed a significant decline in memory performance over time. In the second interference task, both groups showed a similar pattern of performance decline as the number of interpolated tones increased, which is consistent with previous studies (Deutsch, 1970b). Whilst the present evidence suggests that adding increasing number of distractor tones did not lead to proportionally greater interference in individuals with amusia, this conclusion is tempered by the different baseline performance levels of the two groups. Gosselin et al. (2009) reported that individuals with amusia showed greater effects of interference compared to controls when performance levels were similar, but this held only for a fixed number of distractor tones (6 tones). It remains to be determined whether the pattern of similar decline in amusics and controls in response to increasing numbers of interpolated tones persists when overall performance levels are similar. For the purposes of such future research, our findings and those of Gosselin et al. indicate that matching performance in this way requires the use of either different interpolated time intervals for both groups (i.e., shorter time delays for amusics) and/or pitch intervals between S and C tones that exceed 2 tones.

The unexpected between-group difference in the baseline condition of both tasks (time task: no delay; interference task: no intervening tones) is worthy of comment. Individuals with amusia have been shown to have elevated thresholds for the detection of a pitch change (Foxton et al., 2004). However, the interval used in the present experiment was approximately twice the size of the average pitch detection thresholds of Foxton et al.^1^ We propose two alternative theories to explain why individuals with amusia performed more poorly than anticipated in the two baseline conditions.

### Task demands

The present tasks require participants to say “different” to a C tone only when they are convinced a change has occurred. In this situation, individuals with amusia may be more likely than controls to report perceiving no difference, either because their criteria for responding “different” are higher than control participants or because they lack the conscious access to the knowledge that would allow them to make a decision (Peretz, Brattico, Jarvenpaa, & Tervaniemi, 2009). The high proportion of misses compared to false alarms in the time task confirm that individuals with amusia were more likely to say “same” in the present tasks (see Table 4). A similar pattern of errors can be observed in the data from the interference task (see Table 5)

**Table 4.** Group Errors in the Time Task.

		Misses					False alarms				
		0 s	1 s	5 s	10 s	15 s	0 s	1 s	5 s	10 s	15 s
Control	μ	0,53	0,47	1	0,88	0,82	0,29	0	0	0,12	0,59
	σ	1,28	0,80	1,66	1,11	1,29	0,59	0	0	0,33	1,06	
Amusic	μ	**4,12**	**3**	**4,12**	**4,65**	**4,18**	0,82	0,12	0,41	1,18	1,76
	σ	4,14	2,87	3,71	3,50	3,68	1,91	0,33	0,87	1,70	1,95	

*Note*. Table shows the proportion of misses and false alarms for all five time delay conditions.

**Table 5.** Group Errors in the Interference Task.

		Misses					False alarms				
		0 s	2 s	4 s	6 s	8 s	0 s	2 s	4 s	6 s	8 s
Control	μ	0,13	1,8	4	5	4,67	0,2	0,53	1,73	1,6	1,47
	σ	0,35	2,01	2,7	2,65	1,95	0,41	0,92	1,83	1,35	1,19
Amusic	μ	**4,25**	**5,5**	**8,13**	**8,69**	**7,25**	0,69	1,81	2,38	2,19	2,89
	σ	2,49	1,51	2,03	2,09	2,08	1,25	1,17	1,75	1,6	2,13

*Note*. Table shows the proportion of misses and false alarms for all five tone interference conditions.

### Problems with rapid auditory temporal processing (RATP)

Another unexpected finding was the improvement in performance seen in many individuals with amusia between the first two conditions of the time task (no gap vs. a one second gap). Individuals who found the no gap condition particularly difficult reported that the stimuli were too fast to compare effectively. Problems with rapidly presented auditory material have also been reported in other auditory developmental disorders such as specific language impairment (SLI) and dyslexia (Bishop, 2007).

The rapid auditory temporal processing (RATP) theory suggests that difficulty with the resolution of immediately adjacent sounds results in reduced ability to distinguish auditory cues. Bishop (2007) presented ERP data from a number of studies to suggest that SLI and dyslexic children may be especially susceptible to effects like backward masking when auditory stimuli are presented in rapid succession (with inter-stimulus intervals of less than 1s). It is not our intention to make a direct comparison of language/literacy disorders and amusia here. However, since both conditions are linked to the development of the auditory processing mechanisms, it is possible that RATP difficulties are common to both populations, and across language and music stimuli. In support of this suggestion, evidence has shown that SLI and dyslexic children also demonstrate RATP problems when required to distinguish tones that are presented with inter-stimulus intervals ranging from 10 ms up to 600 ms (Bishop, 2007). It may be that a subgroup of individuals with amusia also suffers from RATP difficulties. General auditory processing problems already attributed to some tune deaf individuals include gap detection and estimates of sound duration (Jones, Zalewski, Brewer, Lucker, & Drayna, 2009). Potential implications of an RATP problem in the present time task might include perceiving the tones in the 0 s trials as continuous, even though they had distinct onset and offset amplitude envelopes.

The between-group differences seen in baseline conditions have a bearing on our interpretation of the findings in the time task, where the different gradients of performance decrement over time for the two groups could be interpreted as reflecting a different starting level. To address this possibility, a post-hoc analysis of the data was carried out in order to compare performance when there was no significant difference between the performance levels of the two groups in the no-gap condition. This involved the removal of the data from 6 individuals with amusia whose scores were on average 1.8 *SD* below the rest of the group. The interaction between group and time delay remained significant in this analysis, *F*(3.05, 79.41) = 4.2, *p* = .008, η_p_^2^ = .14, suggesting that even when baseline group performances at 0 s are equivalent (controls = 0.93 and amusics = 0.83: *U* = 72.5, *p* = .25) individuals with amusia show a more significant decrement in performance as a result of increasing time delay between S and C tone. Therefore, the present data suggest that representations of pitch in memory are weaker in individuals with amusia compared to controls.

The results of these two experiments have highlighted the difficulties inherent to the comparison of pitch memory in amusics versus controls. Nevertheless, the current study has shown that the pitch representations of amusics are more prone to decay over time, suggesting fragile storage and/or retention of pitch sounds in memory. Whether these deficits emerge from, or are ancillary to the previously reported elevated pitch discrimination thresholds is not presently known; however, the findings contribute to a growing literature that considers amusia to be more than a deficit of fine grained pitch processing.

## Footnotes

1 No average is reported in the study but the relevant figure shows all but one of the amusics (*N* = 9) have a threshold below 1.5 semitones: One amusic is around 9. Hence we estimate the average would have been 1 semitone or below.

## Appendix A

### Rating scale used to measure musical experience

Please have a look at the following scheme and let me know which category best fits your level of musical expertise:

1) I am a professional musician, meaning that I earn a living by performing music.

2) I am a serious amateur musician, meaning that I reached a high standard at an earlier stage of life and I still keep my hand in, playing several times a year.

3) I was a serious amateur musician - I reached a high standard at an earlier stage of life but I no longer play.

4) I had musical training as a child but I gave up after [ ] years (PLEASE INSERT NUMBER OF YEARS YOU PLAYED)

5) I have never had any musical training.

6) I have not received training on a musical instrument but I am involved with music in a different capacity e.g., I am a DJ/sound engineer/other (PLEASE SPECIFY).
